# Axon Diameter Mapping From Myelin Water Diffusion MRI

**DOI:** 10.1109/TMI.2026.3664328

**Published:** 2026-06

**Authors:** Hong-Hsi Lee, Kwok-Shing Chan, Dmitry S. Novikov, Els Fieremans, Susie Y. Huang

**Affiliations:** Department of Radiology, Athinoula A. Martinos Center for Biomedical Imaging, Massachusetts General Hospital, Charlestown, MA 02129 USA, and also with the Harvard Medical School, Boston, MA 02115 USA; Department of Radiology, Athinoula A. Martinos Center for Biomedical Imaging, Massachusetts General Hospital, Charlestown, MA 02129 USA, and also with the Harvard Medical School, Boston, MA 02115 USA; Center for Advanced Imaging Innovation and Research, New York, NY 10016 USA, and also with the Department of Radiology, New York University School of Medicine, New York, NY 10016 USA; Center for Advanced Imaging Innovation and Research, New York, NY 10016 USA, and also with the Department of Radiology, New York University School of Medicine, New York, NY 10016 USA; Department of Radiology, Athinoula A. Martinos Center for Biomedical Imaging, Massachusetts General Hospital, Charlestown, MA 02129 USA, and also with the Harvard Medical School, Boston, MA 02115 USA

**Keywords:** Diffusion MRI, axon diameter, myelinated axon, Monte Carlo simulations, microstructure imaging

## Abstract

Probing diffusion in myelin water using diffusion-weighted $T_{1}-/T_{2}$-selective MRI acquisitions enables noninvasive measurement of myelinated axon diameter. Its application for in vivo measurements requires numerical verification through diffusion simulations. Here, we propose the theory of myelin water diffusion as measured with a diffusion MRI pulse sequence with wide gradient pulses using the Gaussian phase approximation. We establish its applicability to axonal diameter mapping via Monte Carlo simulations in either infinitely thin cylindrical surfaces or concentric cylindrical shells of finite thickness, mimicking the micro-geometry of myelin sheaths. The estimated diameters are shown to be weighted more toward outer than inner calibers. Simulation results evaluate the theory of myelin water diffusion and axon diameter estimation using spherical mean diffusion signals, demonstrating its applicability at signal-to-noise ratio above 20 on the Connectome 2.0 MRI scanner equipped with maximum gradient strength of 500 mT/m and slew rate of 600 T/m/s. Measuring restricted diffusion of myelin water in-between myelin sheaths using diffusion MRI allows one to measure myelinated axon diameters in vivo. The protocol can potentially be adapted for clinically available high-gradient performance scanners.

## I. Introduction

**D**IFFUSION MRI (dMRI) is sensitive to the characteristics of biological tissues at the cellular level, enabling the noninvasive quantification of relevant tissue microstructural features [1], [2], such as axonal diameter [3], [4], [5], [6], [7], [8], [9], [10], and aiding in the characterization of pathological alterations in white matter observed in conditions ranging from amyotrophic lateral sclerosis [11], [12] to multiple sclerosis [6], [7], [13], [14].

In the brain white matter, the MRI signal originates from multiple compartments, including intra- and extra-axonal water and myelin water [15], i.e., water in-between myelin sheaths. Compared with intra- and extra-axonal water with transverse relaxation times $T_{2}>60\;ms$ and longitudinal relaxation times $T_{1}=1-1.5\;s$ for in vivo human brain tissue [16], [17], [18], [19], myelin water has short $T_{2}$ values (10)–20 ms), short $T_{1}$ values (100–250 ms), and a relatively small contribution to the overall dMRI signal [19], [20], [21], [22]. Conventionally, in vivo imaging of human axonal diameter using echo-planar-imaging based dMRI is sensitive to intra- and extra-axonal water due to the application of long echo times TE ~ 60–80 ms to accommodate the length of typical diffusion gradient pulses and imaging echo trains [6], [7], [8], [9], [10], [23], [24]. In this case, the myelin water signal $∼exp\left(-TE/T_{2}\right)$ may almost entirely decay away due to its short $T_{2}$ value. Further disentanglement of intra- (restricted diffusion) and extra-axonal water (hindered diffusion) requires the application of very strong diffusion gradients with strong diffusion weightings (*b*-values). For in vivo human brain imaging, such measurements may only be practically achievable on high-gradient MRI scanners equipped with maximal gradient strengths $G_{\text{max}}$ of 300 mT/m or higher [25], [26], [27], [28], [29].

The difference in $T_{1}$ and $T_{2}$ values in axonal water and myelin water enables the selective probing of diffusion in myelin water by applying $T_{1}$- and $T_{2}$-selective acquisitions [20], [33], [34] or magnetization transfer (MT) [35] with dMRI. Stanisz et al. estimated diffusion properties of short and long $T_{2}$ components by the using pulsed-gradient (PG) multi-spin-echo CPMG (Carr-Purrell-Meiboom-Gill) sequence in ex vivo bovine optic nerve and showed that the diffusion of the short $T_{2}$ component related to myelin water was less anisotropic than axonal water [33]. Similarly, Peled et al. used a pulsed gradient stimulated echo followed by a CPMG sequence in ex vivo frog sciatic nerve and showed that the restricted diffusion of the short $T_{2}$ component transverse to axons could be explained by a biophysical model of myelin membranes as a series of impermeable concentric cylinders [34]. Andrews et al. applied double-inversion-recovery (DIR) diffusion-weighted CPMG in ex vivo toad sciatic nerve to measure diffusion signals selectively from the short $T_{2}$ component associated with myelin water, whose apparent diffusion coefficients (ADC) were 0.37 and 0.13 *μ*m^2^/ms along and transverse to the nerve, respectively [20]. Avram et al. performed MT-prepared stimulated echo diffusion tensor imaging to provide differential sensitivity to the myelin water signal in the living human brain and observed that myelin water had relatively low radial diffusivity ~ 0.1 *μ*m^2^/ms and relatively high fractional anisotropy ~ 0.9 in the splenium of the corpus callosum, corresponding to a myelin water axial diffusivity & 0.6 *μ*m^2^/ms [35].

The selective acquisition of diffusion signals from myelin water enables the quantification of tissue microstructure related to myelin sheaths. For example, the long $T_{1}$ components related to intra- and extra-axonal water can be saturated by using the inversion recovery sequence [36], and the very long $T_{1}$ component from cerebrospinal fluid can be saturated by using a very short repetition time; in this way, diffusion signal is only contributed by myelin water without partial volume effect. The short $T_{2}$ value of myelin water predictably leads to images with very low signal-to-noise ratio (SNR), which can be mitigated by using a large voxel size, averaging over repeated measurements and multiple gradient directions for spherical mean signals [37], [38], and applying advanced denoising algorithms [39], [40], [41].

Recently, Canales-RodrÍguez et al. proposed to approximate myelin water diffusion as diffusion in a series of infinitely thin concentric cylindrical surfaces and tested the applicability of axonal diameter estimation based on the functional form of myelin water radial diffusivity in the narrow-pulse limit of pulsed gradient sequences [31]. The theory was evaluated by using Monte Carlo simulations of myelin water diffusion in either a series of infinitely thin concentric cylindrical surfaces or a spirally wrapped surface, emulating the realistic geometry of myelin sheaths. They showed that the estimated axonal diameter was correlated with the ground truth values with a non-trivial degree of bias, especially when a high intrinsic diffusivity of myelin water (0.8 *μ*m^2^/ms) was used in simulations. This bias was potentially due to the narrow-pulse approximation in the functional form of myelin water radial diffusivity in biophysical modeling.

To achieve more accurate and precise axonal diameter mapping (ADM) via myelin water diffusion, here we derive the theory of myelin water radial diffusivity for wide-pulse pulsed gradient sequences using the Gaussian phase approximation [2], [23], [42], [43], [44], [45]. Furthermore, we evaluate the resolution limit of axonal diameter estimation, i.e., the minimal detectable diameter, for a given SNR and dMRI protocol [46]. Finally, we perform Monte Carlo simulations of myelin water diffusion in 50 artificially generated axons with multiple cylindrical shells of finite thickness and permeable myelin lamellae to evaluate the theory and demonstrate the applicability of ADM using wide-pulse pulsed gradient sequences.

## II. Theory

### A. Narrow-Pulse Pulsed Gradient Sequence

The myelin sheath has a concentric lamellar structure with a radial periodicity $l_{m}\approx 12\;nm$ in $N_{m}∼25-40$ loops around an axon [47]. To calculate the myelin water radial diffusivity (RD), we approximate the diffusion in-between two adjacent lamellae as one-dimensional Gaussian diffusion around a circle of radius $r=r_{j}+\frac{1}{2}l_{m}$ and zero thickness, and assume no exchange between the two concentric circles of different radii, i.e., $r_{j}$ and $r_{j+1}=r_{j}+l_{m}$. Calculating the second order displacement cumulant transverse to a circle, we obtain the time-dependent diffusivity transverse to a 1-dimensional ring of radius r in the narrow-pulse limit [31]:

(1) $$D^{⊥}(t)\equiv \frac{\left\langle x^{2}\right\rangle }{2t}$$

(2) $$=\frac{r^{2}}{2t}\left(1-e^{-t/t_{c}}\right)$$

with correlation time $t_{c}\equiv r^{2}/D_{0}$ and intrinsic diffusivity $D_{0}$ of myelin water. At very long time ($t\gg t_{c}$), the RD is approximated by

(3) $$D^{⊥}(t)≃\frac{r^{2}}{2t},\;\;t\gg t_{c}.$$

In general, the radial signal in each MR voxel is the volume-weighted sum of compartmental contributions. In each voxel, the myelin water of myelinated axons can be considered as a collection of multiple myelin layers (space between two adjacent lamellae), approximated by circles in different radii. Given that the water volume of each myelin layer is roughly $2\pi r\cdot l_{m}$, proportional to r, the volume-weighted sum of radial MR signal $exp\left(-bD^{⊥}\right)$ becomes [9], [23]

$$S^{⊥}=\frac{\left\langle r\cdot e^{-bD^{⊥}}\right\rangle }{\left\langle r\right\rangle }.$$

For axonal myelin layers in small radii, the radial signal attenuation can be approximated by $exp\left(-bD^{⊥}\right)≃1-bD^{⊥}$ due to $bD^{⊥}\ll 1$, leading to

$$\begin{array}{l} S^{⊥}\;≃\frac{\left\langle r\cdot \left(1-bD^{⊥}\right)\right\rangle }{\left\langle r\right\rangle }=1-b\frac{\left\langle rD^{⊥}\right\rangle }{\left\langle r\right\rangle }\\ \;≃\exp\left(-b\frac{\left\langle rD^{⊥}\right\rangle }{\left\langle r\right\rangle }\right). \end{array}$$

In other words, for small $bD^{⊥}$, the radial signal attenuation averaged over all myelin layers inherits the same exponential functional form with a volume-averaged RD, i.e., $\left\langle rD^{⊥}\right\rangle /\langle r\rangle$.

Following this idea, in narrow-pulse PG sequence, we can volume-average the RD at long times (3) by taking $r^{2}\to r_{\text{eff,NP}}^{2}$ [23], [31]:

(4) $$r_{eff,NP}\equiv {\left(\frac{\left\langle r^{3}\right\rangle }{\left\langle r\right\rangle }\right)}^{1/2},$$

where the subscript “NP” denotes the narrow-pulse pulsed gradient sequence. The effective radius provides a histological reference to be compared with results from fitting the equation to the dMRI data. However, the effective radius $r_{\text{eff,NP}}$ (4) relies on the assumption of small $bD^{⊥}$, which may not apply to myelin layers with large radii due to $D^{⊥}\propto r^{2}$.

Alternatively, a naive volume-weighted average (VWA) of myelin layer radii can be another candidate to interpret the dMRI-estimated radii [31],

(5) $$r_{vwa}=\frac{\left\langle r^{2}\right\rangle }{\left\langle r\right\rangle },$$

which is an empirical guess for the interpretation.

### B. Wide-Pulse PG

For a wide-pulse pulsed gradient sequence of inter-pulse duration ∆ and pulse duration δ, Canales-RodrÍguez et al. extended the narrow-pulse solution (2) by defining diffusion time t=Δ+δ [31]. However, this extension for wide pulse sequence relies on assumptions of unrestricted Gaussian diffusion and small δ/∆ ratio [48], which may not apply to restricted diffusion in myelin water and actual diffusion MRI protocols. Instead, we derive the apparent diffusivity transverse to a 1-dimensional circle of radius r and zero thickness using Gaussian phase approximation (Appendix A), given by

(6) $$\begin{array}{l} D^{⊥}(\Delta ,\delta )=\frac{r^{2}}{2(\Delta -\delta /3)}\cdot \frac{t_{c}^{2}}{\delta^{2}}\cdot \left[2\frac{\delta }{t_{c}}-2+2e^{-\Delta /t_{c}}\right.\\ \;\left.+2e^{-\delta /t_{c}}-e^{-(\Delta -\delta )/t_{c}}-e^{-(\Delta +\delta )/t_{c}}\right]. \end{array}$$

In the wide-pulse limit ($\delta \gg t_{c}$), the apparent radial diffusivity is approximated by

(7) $$D^{⊥}(\Delta ,\delta )≃c\cdot \frac{r^{4}}{D_{0}}\cdot \frac{1}{\delta (\Delta -\delta /3)},\;\delta \gg t_{c}$$

with c=1, sharing the same functional form of Neuman’s solution with c=7/48 for diffusion inside a cylinder in the wide-pulse limit [43]. In other words, the radial signal attenuation of myelin water is inherently larger than that of intra-axonal water under the same diffusion protocol. Similar to the discussion in Section II-A, considering MR contributions of multiple myelin layers within a voxel, we need to calculate their volume-weighted sum of RD. Given that the water volume of each myelin layer is proportional to r, the volume-weighted sum of RD in the wide-pulse limit (7) is re-weighted by taking $r^{4}\to r_{\text{eff,WP}}^{4}$ [23]:

(8) $$r_{eff,WP}={\left(\frac{\left\langle r^{5}\right\rangle }{\left\langle r\right\rangle }\right)}^{1/4},$$

where the subscript “WP” denotes the wide-pulse pulsed gradient sequence. This effective radius definition relies on the assumption of small $bD^{⊥}$, which may not apply to myelin layers with large radii due to $D^{⊥}\propto r^{4}$.

### C. MyCaliber Model for ADM

We approximate myelin water diffusion in myelin sheaths of each axon segment as an axisymmetric diffusion tensor [31] with axial diffusivity (AD) $D^{\|}$ and RD $D^{⊥}$ for narrow-pulse (2) or wide-pulse (6) pulsed gradient sequence. The diffusion along the axon segment is assumed to be Gaussian with no diffusivity time-dependence, whereas the diffusion transverse to the myelin segment is restricted and non-Gaussian with diffusivity time-dependence.

To factor out fiber orientation dispersion of myelinated axons, we directionally average dMRI signals at each b-value using the spherical mean technique [8], [9], [37], [38], yielding the normalized spherical mean signal

(9) $$\overset{‾}{S}\left(b\right)=\exp\left(-bD^{⊥}\right)\cdot h\left(A\right),$$

with the diffusion weighting (b-value)

(10) $$\begin{array}{l} \;b=\gamma^{2}G^{2}\delta^{2}(\Delta -\delta /3),\\ h\left(y\right)=\int_{0}^{1}\;d\xi e^{-y\xi^{2}}=\sqrt{\frac{\pi }{4}}\cdot \frac{\mathrm{erf}\left(\sqrt{y}\right)}{\sqrt{y}}, \end{array}$$

and

(11) $$A=b\left(D^{\|}-D^{⊥}\right).$$

By fitting (9) to the spherical mean signal of myelin water, we can estimate the myelin water RD $D^{⊥}$ and the subsequent axonal diameter 2r by using (6) for wide-pulse pulsed gradient sequences. We call the ADM technique *My*elinated axon *Caliber* estimation (MyCaliber).

Alternatively, the estimated RD can be translated into an axon diameter estimate by using the extended narrow-pulse solution (2) with t=∆+δ, as suggested in [31]. We will compare our MyCaliber model with this approach.

### D. Resolution Limit of Axon Radius Estimation

To reliably estimate the axon radius, the diffusion signal decay (usually the largest at the highest b-value) must be larger than the noise level. Based on the spherical mean signal (9) and myelin water RD for narrow-pulse (2) or wide-pulse (6) pulsed gradient sequence, the larger the radius r, the greater the signal decay. In other words, comparing the signal decay and noise level suggests a minimally detectable axon radius, i.e., the resolution limit of axon radius estimation [46].

For narrow-pulse pulsed gradient sequence, the resolution limit of the effective radius (4) is (Appendix B) [46], [49]

(12) $$r_{min,NP}=\sqrt{\frac{2t\overset{‾}{\sigma }}{b}}\cdot h^{-1/2}(A),$$

with $\overset{‾}{\sigma }=z_{\alpha }/(SNR\;\left.\sqrt{n_{g}}\right),$ $z_{\alpha }=1.64$ for a one-sided test at 5% significance level, and the number $n_{g}$ of gradient directions per b-shell. Here we approximate $A≃bD^{\|}$ in (11) since the myelin water $D^{⊥}$ is much smaller than its $D^{\|}≃D_{0}∼0.8\;\mu m^{2}/ms$ at diffusion time t∼10ms for axons of radii ~ 1 *μ*m (Appendix B). Furthermore, we extended the narrow-pulse resolution limit (12) for finite pulse width by substituting t=Δ+δ.

Similarly, for wide-pulse pulsed gradient sequence, the resolution limit of the effective radius (8) is (Appendix B) [46], [49]

(13) $$r_{min,WP}={\left(\frac{D_{0}\overset{‾}{\sigma }}{\gamma^{2}G^{2}\delta }\right)}^{1/4}\cdot h^{-1/4}(A),$$

where we approximate $A≃bD^{\|}$ in (11) since the myelin water $D^{⊥}$ is much smaller than its $D^{\|}≃D_{0}∼0.8\;\mu m^{2}/ms$ at Δ=13 ms and δ=6ms for axons of radii ~ 1 *μ*m (Appendix B). The (13) shows that the stronger the diffusion gradient strength G, the smaller (better) the resolution limit $r_{\text{min,WP}}$.

## III. Methods

### A. Diffusion Simulations of RD: One-Dimensional Circle

We performed Monte Carlo simulations of diffusion on an infinitely thin cylindrical surface (one-dimensional circle) of radius r=1,2, and 3 *μ*m. For each simulation, we applied 1×10^5^ walkers randomly hopping along the circle with a step angle [31]

δθ=δx/r,

where the step size along the circle is $\delta x=\sqrt{2D_{0}\delta t}$, the time step is $\delta t=1\times 10^{-3}\;ms$, and the intrinsic diffusivity is $D_{0}=0.8\;\mu m^{2}/ms$.

For narrow-pulse pulsed gradient sequences, we calculated RD (1) by using diffusion displacement cumulants $\left\langle x^{2}\right\rangle$ transverse to the circle at diffusion times t=1-25ms. Monte Carlo simulations were implemented in MATLAB and performed on an AMD EPYC 7313 3.0 GHz processor [54]. The total calculation time was 12 min.

For wide-pulse pulsed gradient sequences, we calculated diffusion signals $\left\langle e^{i\phi }\right\rangle$ from diffusional phase ϕ=γ∫G⋅xdt at six b-values = 1–6 ms/*μ*m^2^, four gradient directions transverse to the circle, five pulse widths δ=6-10ms, and inter-pulse durations $\Delta =\delta +t_{RF180}$ to accommodate a 180° radio-frequency (RF) refocusing pulse of $t_{RF180}=5\;ms$ in width. The gradient strength G was calculated based on the b-value definition (10). RD at each (Δ,δ) combination was calculated by using the cumulant expansion up to the fourth order of b-value.

### B. Diffusion Simulations of RD: Two-Dimensional Ring

The cylindrical surface (or one-dimensional circle) has zero thickness, and the cylindrical shell (or two-dimensional ring) has a finite thickness of myelin layer. Strictly speaking, the finite thickness of cylindrical shell might affect diffusion measurements, and thus we evaluated the effect of layer thickness on myelin water diffusion in simulations.

We performed Monte Carlo simulations of diffusion within a cylindrical shell (2-dimensional ring) of finite thickness $l_{m}=12\;nm$ and inner radius $r_{i}=1,\;2$, and 3 *μ*m. For each simulation, we applied 1×10^5^ random walkers diffusing in a two-dimensional space within the myelin layer, with a step size $\delta x=\sqrt{4D_{0}\delta t}$, a time step $\delta t=1\times 10^{-6}\;ms$, and an intrinsic diffusivity $D_{0}=0.8\;\mu m^{2}/ms$. The random walker experienced an elastic collision when encountering the membrane. Monte Carlo simulations were implemented in CUDA C++ and performed on an NVIDIA A40 GPU core [54]. The total calculation time was 40 min.

For narrow-pulse pulsed gradient sequences, RD was calculated by using diffusion displacement cumulants transverse to the ring at diffusion times t=1-25ms. For wide-pulse pulsed gradient sequences, RD was calculated by using diffusion signals from diffusional phase of the same protocol in Section III-A.

### C. Diffusion Simulations of ADM: Three-Dimensional Shells

We created 50 axons composed of $N_{m}$ concentric cylindrical shells of finite thickness $l_{m}=12\;nm$ [47]. We varied the inner diameter from $2r_{i}=0.2-10\;\mu m$ with an increment of 0.2 *μ*m. The number of myelin layers $N_{m}$ was calculated based on a relation of inner diameter $2r_{i}$ and g-ratio (ratio of inner to outer diameters) in previous histological studies [50], [51], [52], [53], which suggested a log-linear functional form

(14) $$N_{m}=C_{0}+C_{1}\cdot \left(2r_{i}\right)+C_{2}\cdot ln\left(2r_{i}\right)$$

with $C_{0}l_{m}=0.35\;\mu m,$ $C_{1}l_{m}=0.006$, and $C_{2}l_{m}=0.024\;\mu m$.

We performed Monte Carlo simulations of diffusion by applying 1×10^4^ random walkers per axon, i.e., 5×10^5^ random walkers in total, diffusing in a three-dimensional space within_√_ each myelin layer with a step size $\delta x=\sqrt{4D_{0}\delta t}$ transverse to the axon, a step size $\delta z=\sqrt{2D_{0}\delta t}$ along the axon, a time step $\delta t=1\times 10^{-6}\;ms$, and an intrinsic diffusivity $D_{0}=0.8\;\mu m^{2}/ms$. Myelin lamellae between myelin layers were modeled as permeable membranes of permeability $\kappa =6.7\times 10^{-3}\;\mu m/ms$ based on an in vivo human brain study using the pulsed magnetization transfer MR sequence [55]. The permeation probability across the membrane was given by [54] and [56]

$$P\equiv \frac{P_{0}}{1\;+\;P_{0}},\;P_{0}\equiv \frac{\pi }{4}\cdot \frac{\kappa \delta x}{D_{0}}.$$

Given that the surface-to-volume ratio of myelin water was much larger than that of intra- and extra-axonal water (Appendix C), we only considered water exchange between adjacent myelin layers and ignored exchange between myelin water and (intra- and extra-) axonal water.

We calculated diffusion signals based on the protocol with eight b-values = 0.05, 0.35, 0.8, 1.5, 2.4, 3.45, 4.75, and 6 ms/*μ*m^2^, 64 gradient directions g per b-shell, a pulse width δ=6ms, and an inter-pulse duration Δ=13ms. Such diffusion protocols can be implemented on the human Connectome 2.0 scanner, equipped with a maximum gradient strength $G_{\text{max}}$ of 500 mT/m and a maximum gradient slew rate of 600 T/m/s [28], [29]. Similar diffusion protocols of the same b-values can be achieved on other high gradient performance MR systems, such as the human Connectome 1.0 scanner with a $G_{\text{max}}=300\;mT/m$ (δ=9ms,Δ=16ms) [25], [57], and commercially available human scanners (MAGNUS, Impulse, Cima.X) with a $G_{\text{max}}=200\;mT/m$ (δ=12ms,∆=19ms) [27], [58]. Monte Carlo simulations were implemented in CUDA C++ and performed on an NVIDIA A40 GPU core [54]. The total calculation time for all 50 axons was 8 hours.

We combined simulated signals of all 50 axons based on an inner diameter distribution inspired by histology [3], [53], [59], described by the Gamma distribution $\rho \left(r_{i},\kappa ,\vartheta \right)$ with a shape parameter κ and a scale parameter ϑ. We varied the mean of inner diameter κϑ=0.5-2μm, and set up the standard deviation of inner diameter $\sqrt{\kappa }\vartheta$ as half of the mean. This relationship between the mean and standard deviation of inner diameter was previously observed in histology [53], [59].

Rician noise was applied to combined signals S(b,g) at signal-to-noise ratio (SNR) = Inf (no noise), 50, 20, and 10. The Rician noise floor was corrected by using [60]

$$\overset{ˆ}{S}(b,g)=\sqrt{max\left(S^{2}(b,g)-\sigma^{2},0\right)}$$

with the noise floor σ=1/SNR.

We calculated the spherical mean signal $\overset{‾}{S}(b)$ by averaging the combined signals $\overset{ˆ}{S}(b,g)$ over all gradient directions g at each b-value. We fitted MyCaliber model (9) to spherical mean signals of myelin water using nonlinear least squares. We estimated axon diameter 2r using myelin water RD solution (6) for wide-pulse pulsed gradient sequence or extended narrow-pulse solution (2) with t=Δ+δ. The intrinsic diffusivity $D_{0}$ was approximated with the axial diffusivity $D^{\|}$, leading to a two-parameter fit with parameters 2r and $D^{\|}$. Finally, we compared the fitted diameter with the reference from micro-geometries, such as mean diameter 2⟨r⟩, volume-weighted averaged diameter $2r_{vwa}$ in (5), effective diameter of narrow-pulse pulsed gradient sequence $2r_{\text{eff,NP}}$ in (4), and effective diameter of wide-pulse pulsed gradient sequence $2r_{\text{eff,WP}}$ in (8).

### D. Diffusion Simulations in Cylindrical Shells With Caliber Variations and Undulations

In pathological tissues, axons may have caliber variations (beadings) and undulations (micro-dispersion along axon) [61], [62], [63], [64], potentially resulting in non-trivial effects on diffusion parameter estimations. For example, intra-axonal diffusivity along axons decreased with the presence of caliber variations and undulations, and intra-axonal diffusivity transverse to axons increased with the presence of undulations, leading to biases in axon diameter estimations from intra-axonal water diffusion [49], [65], [66], [67], [68].

To demonstrate these effects, we generated concentric cylindrical shells of finite thickness $l_{m}=12\;nm$ and total axon length of 60 *μ*m with caliber variations and undulations, controlled by coefficient of variation of inner radius CV(r) (standard deviation divided by mean) and undulation amplitude $w_{0}$, respectively [68]. The bead shape was modulated as a Gaussian kernel, and the undulation of axon skeleton was modeled as a sinusoidal waveform. We created four cylindrical shells by varying the two shape parameters as $\left(CV(r),w_{0})\;=\;(0,\;0),\;(0,\;0.5\;\mu m),\;(0.2,\;0\right)$, and (0.2, 0.5 *μ*m). Other shape parameters included the mean bead distance of 5 *μ*m, standard deviation of bead distance of 2.5 *μ*m, bead width of 5 *μ*m, mean cross-sectional area of intra-axonal space in $\pi \times (1\;\mu m{)}^{2}$, and undulation wavelength of 20 *μ*m. The values of shape parameters were chosen based on histological results in electron microscopy data of the mouse brain corpus callosum [53], [67].

We performed Monte Carlo simulations of diffusion within each single-layer cylindrical shell with varying caliber variations and undulations. Instead of transforming the axon shape into watertight meshes or pixelated geometries, the inner and outer membranes of each cylindrical shell was analytically expressed by multiple connecting oblique frustums with a thin height of 0.02 *μ*m and parallel top and bottom faces [56]. For each simulation, we applied 1×10^5^ random walkers diffusing in a three-dimensional space within each cylindrical shell, with a step size $\delta x=\sqrt{6D_{0}\delta t}$, a time step $\delta t=1\times 10^{-6}\;ms$ and an intrinsic diffusivity $D_{0}=0.8\;\mu m^{2}/ms$. Due to the shape complexity, we implemented rejection sampling for impermeable membranes (staying still for one step) when the random walker encountered the membrane [69], [70]. We implemented periodic boundary conditions along the top and bottom edges of each axon, whose top and bottom faces were well aligned during creation. Monte Carlo simulations were implemented in CUDA C++ and performed on an NVIDIA A40 GPU core [54]. The total calculation time for each axon was 30 min.

For narrow-pulse pulsed gradient sequences, AD and RD were calculated by using diffusion displacement cumulants along and transverse to the axon’s main axis at diffusion times t=1-25ms. For wide-pulse pulsed gradient sequences, AD and RD were calculated by using diffusion signals from diffusional phase of the same protocol in Section III-A.

The source codes of all simulations and model fitting are available on https://github.com/Connectome20/MyCaliber.

## IV. Results

### A. Diffusion Simulations of RD

We performed diffusion simulations either on a cylindrical surface (1-dimensional circle, Fig. 2a) or in a cylindrical shell of finite thickness (2-dimensional ring, Fig. 2b) and calculated diffusivity transverse to the cylinder. In both simulations, the simulated RD coincided with solutions of narrow-pulse (2) and wide-pulse (6) pulsed gradient sequences, demonstrating that the RD solutions for infinitely thin cylindrical surfaces were applicable not just to cylindrical surfaces but also to cylindrical shells of infinite thickness when the thickness-to-radius ratio $\left(l_{m}/r_{i}\right)$ is small [30].

### B. Diffusion Simulations of ADM

To test the feasibility of ADM using myelin water diffusion, we created 50 axons in different inner radii $r_{i}$ composed of $N_{m}$ cylindrical shells of the same thickness $l_{m}$ (Fig. 3a). The number of shells was computed based on a log-linear relation (14) observed in histology (Fig. 3b-c) [53]. We performed diffusion simulations in cylindrical shells of the 50 axons, mimicking the water diffusion in-between myelin sheaths of myelinated axons, and calculated diffusion signals based on a diffusion protocol compatible with the capabilities of the human Connectome 2.0 MRI scanner. Simulated signals of 50 axons were combined based on a inner radius distribution, modeled as a Gamma distribution (Fig. 3d), and the Rician noise was applied to combined signals. Under different SNR, we calculated spherical mean signals $\overset{‾}{S}(b)$ (Fig. 4) and fitted the MyCaliber model (9) with wide-pulse RD solution (6) to $\overset{‾}{S}(b)$ for the axon diameter estimation.

MyCaliber model (9) agreed with simulated spherical mean signals from 50 axons of multiple cylindrical shells (Fig. 4). Furthermore, we compared the fitted diameter 2r with four possible interpretations, such as mean diameter 2⟨r⟩, volume-weighted averaged diameter $2r_{\text{vwa}}$ in (5), effective diameter of narrow-pulse pulsed gradient sequence $2r_{\text{eff,NP}}$ in (4), and effective diameter of wide-pulse pulsed gradient sequence $2r_{\text{eff,WP}}$ in (8) (Fig. 5).

Empirically, by using the wide-pulse solution (6), the estimated diameter matched the best with the volume-weighted averaged diameter $2r_{\text{vwa}}$ with the smallest bias (Fig. 5a). At SNR ≥ 50, the estimated diameter demonstrated a consistent positive correlation with the volume-weighted averaged diameter with very small overestimations. Furthermore, at SNR = 20, the estimated diameter coincided with the volume-weighted averaged diameter when $2r_{vwa}\geq 1.5\;\mu m$, as predicted by the resolution limit (13) of wide-pulse pulsed gradient sequence, whereas the estimated diameter was smaller than the volume-weighted averaged diameter (underestimation) when $2r_{\text{vwa}}<1.5\;\mu m$.

In contrast, by using the extended narrow-pulse solution (2) with t=Δ+δ, the estimated diameter was lower than all four possible interpretations of diameters (Fig. 5b). The estimated diameter showed a consistent positive correlations for all four interpretations with noticeable underestimations. In addition, the resolution limit (12) with t=Δ+δ could not predict the smallest detectable axon diameter, below which the model fitting yielded near-zero estimates since diffusion signals of zero diameter and finite diameter were indistinguishable at a given SNR.

### C. Effects of Realistic Axonal Shapes on Diffusion Metrics

To demonstrate the effect of complicated axonal shape on myelin water diffusion, we generated cylindrical shells of realistic axonal shape with caliber variations and undulations and performed Monte Carlo simulations of diffusion in the myelin layer of these axons (Fig. 6a). Simulation results showed that the narrow-pulse radial diffusivity was hardly affected by caliber variations and undulations, and the wide-pulse radial diffusivity was hardly affected by caliber variations and mildly affected by undulations (Fig. 6b). The general trend of radial diffusivity time-dependence in narrow-pulse and wide-pulse sequences was predicted by the proposed wide-pulse solution (6), and the extended narrow-pulse solution (2) with t=Δ+δ had non-trivial bias. For diffusion along axons, axial diffusivity in both narrow-pulse and wide-pulse sequence was slightly lower than the intrinsic diffusivity by less than 4% due to caliber variations and undulations (Fig. 6c).

## V. Discussion and Conclusion

We performed Monte Carlo simulations of diffusion on an infinitely thin cylindrical surface (one-dimensional circle) and in a cylindrical shell of finite thickness (two-dimensional ring) and evaluated the theory of myelin water RD of narrow-pulse (2) and wide-pulse (6) pulsed gradient sequences.

Furthermore, to evaluate the applicability of ADM using myelin water diffusion MRI, we created 50 axons with multiple coaxial cylindrical shells, mimicking the myelin sheaths of myelinated axons. We then performed diffusion simulations in cylindrical shells (myelin layers) of these axons and calculated diffusion signals using diffusional phase based on a diffusion MRI protocol achievable on the human Connectome 2.0 scanner. This diffusion protocol can be adapted for other high gradient performance MR systems with $G_{max}=200-300\;mT/m$. We combined simulated signals of 50 axons based on an inner radius distribution, applied Rician noise to the combined signals, and calculated spherical mean signals for ADM. We fitted the MyCaliber model to simulated spherical mean signals to estimate the axon diameter and compared fitted diameters with effective diameters based on micro-geometries of simulation substrates.

**Fig. 1.**
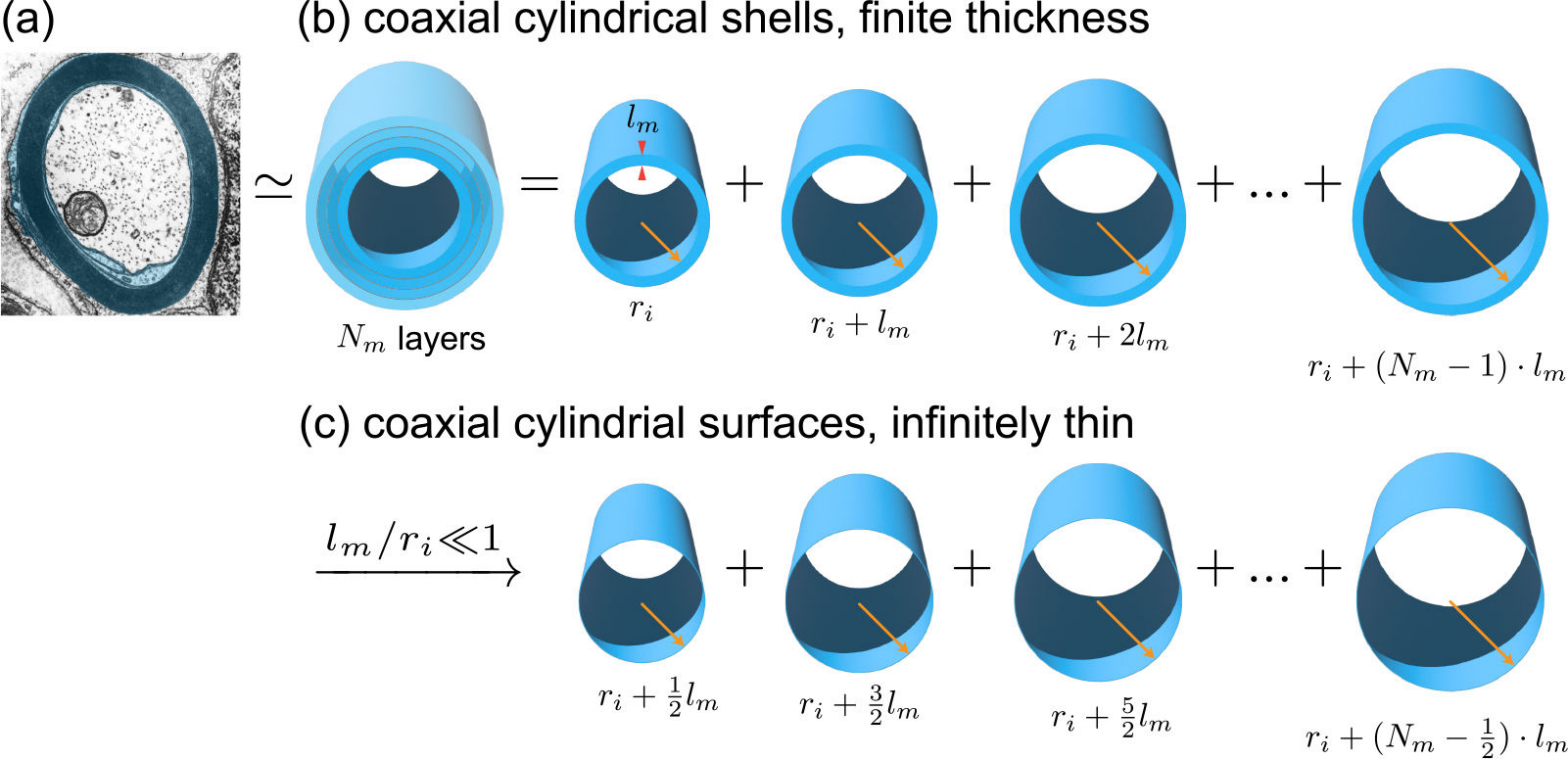
Biophysical model of myelin water diffusion. (a) Diffusion of myelin water in-between myelin sheaths ($N_{m}$ layers) of a myelinated axon (inner radius $r_{i}$) can be considered as (b) diffusion in a collection of cylindrical shells [30] in varying inner radii $r_{i}+(j-1)\cdot l_{m}\left(j=1,2,\ldots ,N_{m}\right)$ and a fixed layer thickness $l_{m}$. (c) Given that the myelin layer thickness $l_{m}∼12\;nm$ is much smaller than the axon inner radius $r_{i}∼1\;\mu m$, this physical picture can be further simplified as diffusion in a collection of cylindrical surfaces [31] in varying radii $r_{i}+\left(j-\frac{1}{2}\right)\cdot l_{m}$ that are infinitely thin, i.e., $l_{m}/r_{i}\ll 1$. Panel (a) is adapted from [32] with the permission of Elsevier.

**Fig. 2.**
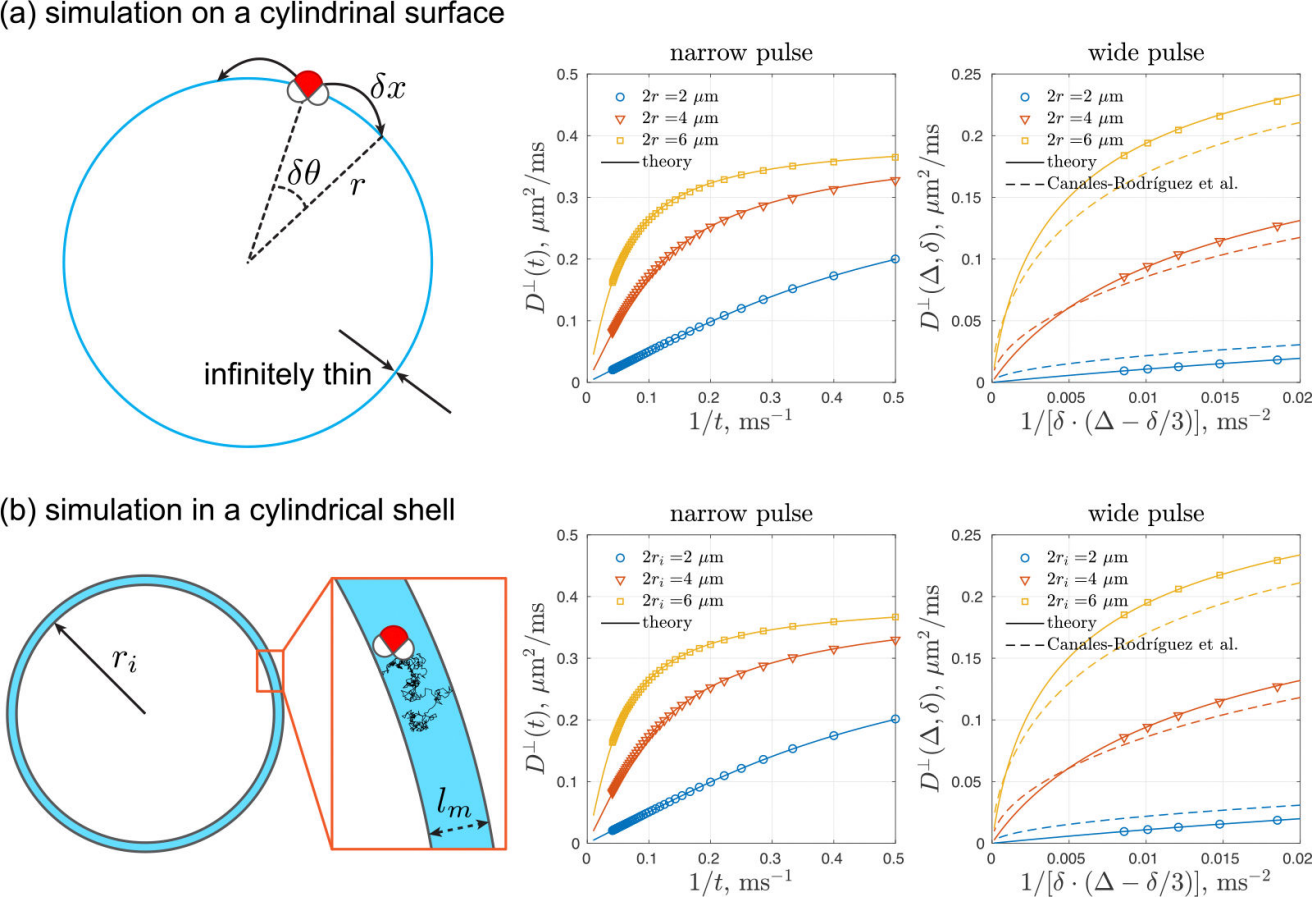
Monte Carlo simulation of radial diffusivity in myelin water. (a) Diffusion simulation on an infinitely thin cylindrical surface can be considered as a random hopping process on a one-dimensional circle in a diameter 2r, with the hopping step size δx and step angle δθ=δx/r. The simulated radial diffusivity (data points), i.e., $D^{⊥}(t)$ for narrow-pulse and $D^{⊥}(\Delta ,\delta )$ for wide-pulse pulsed gradient sequences, matches the theory (solid curves) of myelin water diffusivity transverse to the circle in (2) for narrow-pulse and (6) for wide-pulse pulsed gradient sequences. In contrast, the alternative wide-pulse solution (dashed curves) extended from the narrow-pulse one, i.e., (2) with t=Δ+δ [31], does not agree with simulations well. (b) Diffusion simulation in a cylindrical shell of an inner diameter $2r_{i}$ and a finite thickness $l_{m}=12\;nm$ can be considered as the diffusion in a 2-dimensional ring. Similarly, the simulated radial diffusivity (data points) matches the theory (solid curves).

**Fig. 3.**
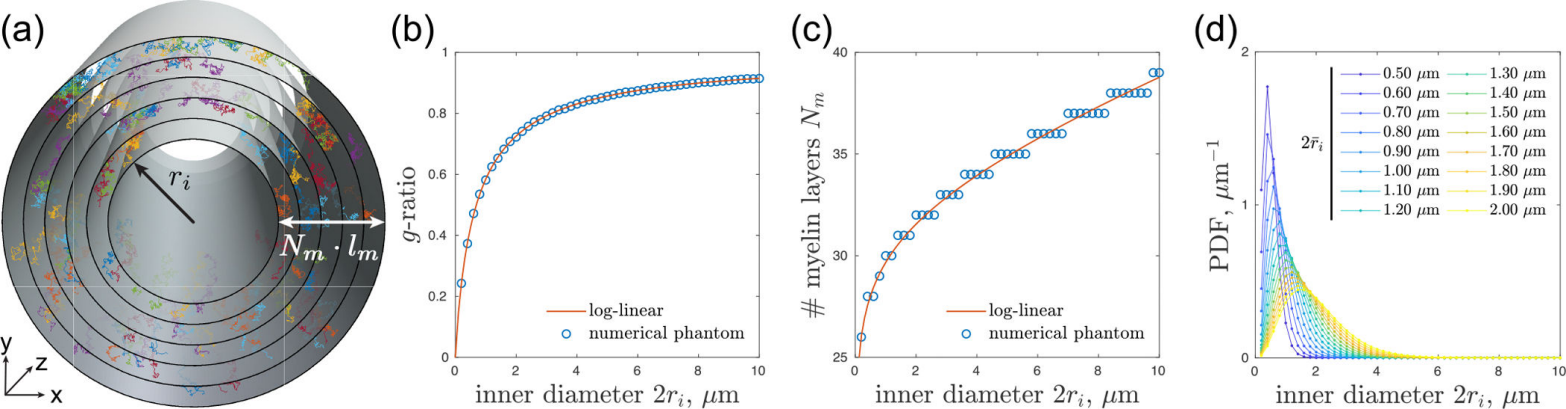
Building numerical phantoms for diffusion simulations of axonal diameter mapping. (a) We created 50 axons mimicking myelinated axons, with inner diameter $2r_{i}=0.2-10\;\mu m$ and $N_{m}$ cylindrical shells of finite thickness $l_{m}=12\;nm$. Here we showed an example of $N_{m}=5$. (b) The number of layers was calculated based on the relation of axon inner diameter and g-ratio in previous histological studies [50], [51], [52], [53], which suggested (c) a log-linear relation between the axon inner diameter and layer number $N_{m}$. (d) Further, we combined simulated signals of each axon based on an axon inner diameter distribution, described by a Gamma distribution with a mean diameter $2{\overset{‾}{r}}_{i}=0.5-2\;\mu m$ and a standard deviation equal to half of the mean.

**Fig. 4.**
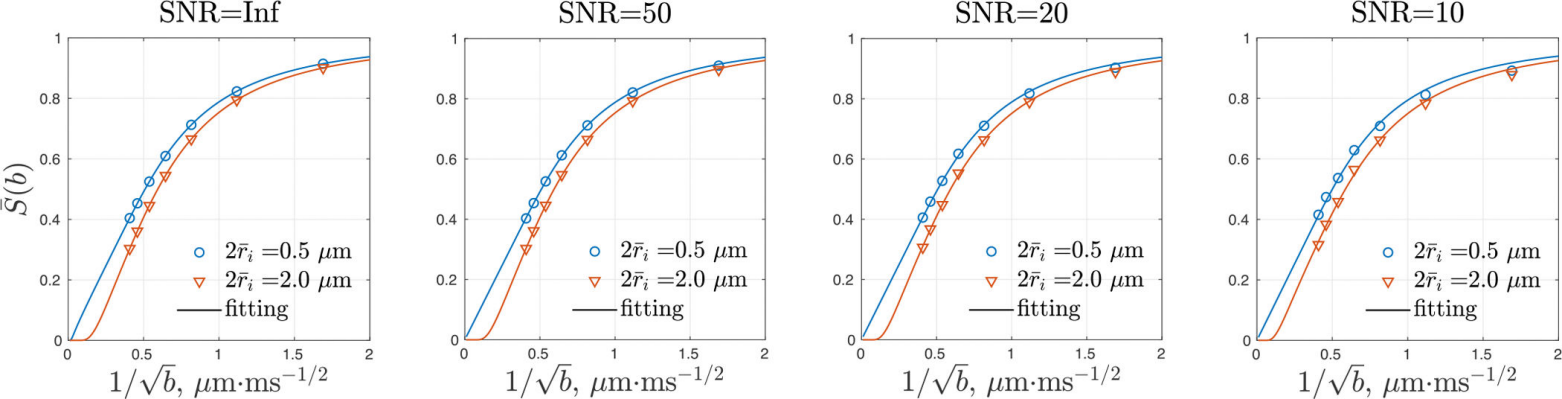
Simulated spherical mean signal $\overset{‾}{S}(b)$ at each b-value. We performed Monte Carlo simulations of diffusion in 50 axons with different axon inner diameter $2r_{i}$ and multiple coaxial cylindrical shells of finite thickness and permeable membranes (Fig. 3). We combined their diffusion signals S(b,g) in each b-value and direction g based on the axon inner diameter distribution (mean diameter $2{\overset{‾}{r}}_{i}=0.5-2\;\mu m$) and applied the Rician noise at SNR = Inf (no noise), 50, 20, and 10. We then calculated the spherical mean signal $\overset{‾}{S}(b)$ at each b-value and fitted MyCaliber model (9) to $\overset{‾}{S}(b)$ to estimate the axon diameter 2r based on the radial diffusivity $D^{⊥}(\Delta ,\delta )$ of the wide-pulse pulsed gradient sequence (6).

**Fig. 5.**
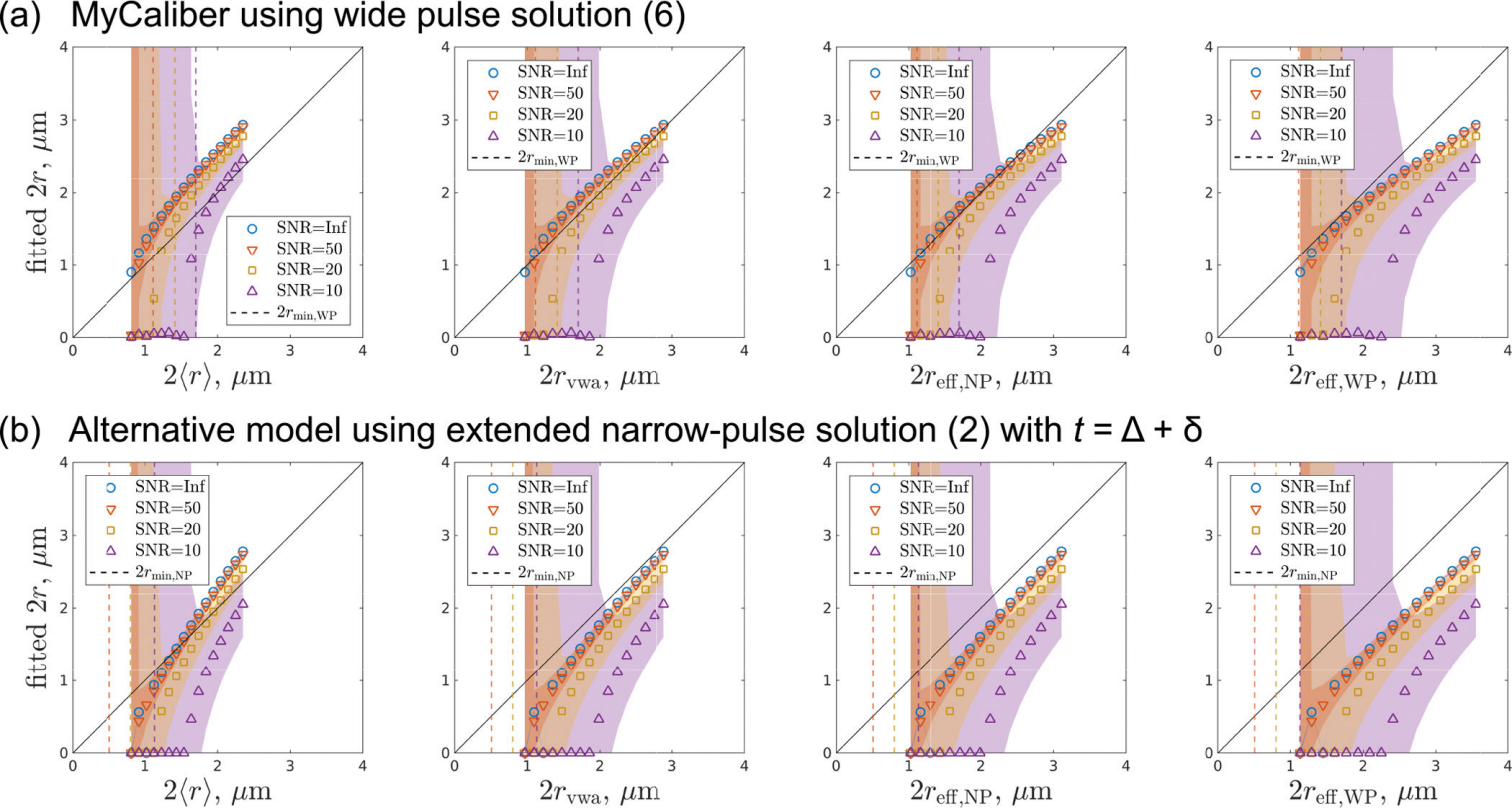
Axonal diameter mapping from myelin water diffusion signals. (a) We performed Monte Carlo simulations of myelin water diffusion in axons of multiple coaxial cylindrical shells with permeable membranes and fitted the MyCaliber model (9) to simulated spherical mean signals at different SNR levels. We estimated axon diameter 2r based on the radial diffusivity of wide-pulse solution (6) and (b) alternative solution extended from the narrow-pulse solution (2) with t=Δ+δ [31]. The estimated diameter was compared with the effective diameter defined in a variety of ways, such as mean diameter 2⟨r⟩, volume-weighted averaged diameter $2r_{vwa}$ (5), effective diameter of narrow-pulse sequence $2r_{\text{eff},NP}$ (4), and effective diameter of wide-pulse sequence $2r_{\text{eff,}\;\text{WP}}$ (8). The shaded area indicates the range of values within one standard deviation of the fitted axon diameter.

Simulation results demonstrated that the fitted diameters matched the best with the volume-weighted averaged diameter $2r_{vwa}$ in (5). At SNR ≥ 20, axonal diameter can be reliably estimated using myelin water diffusion MRI when $2r_{\text{vwa}}\geq 1.5\;\mu m$. The effective diameter of myelin layer in (4) and (8) is calculated by averaging diameters over all myelin layers and is more weighted by the tail of diameter distribution of myelin layer [23], i.e., the larger the diameter, the larger the averaging weights. Therefore, the axon diameter estimated from myelin water diffusion is more toward to the outer than inner calibers (Fig. 1) and should be larger than that estimated from intra-axonal water diffusion related with only inner calibers [3], [4], [5], [6], [7], [8], [9], [10]. Compared with conventional approaches targeting intra-axonal water diffusion, the proposed MyCaliber provided additional tissue parameters, such as outer-weighted axon diameter and myelin water axial diffusivity, that were challenging to estimate.

**Fig. 6.**
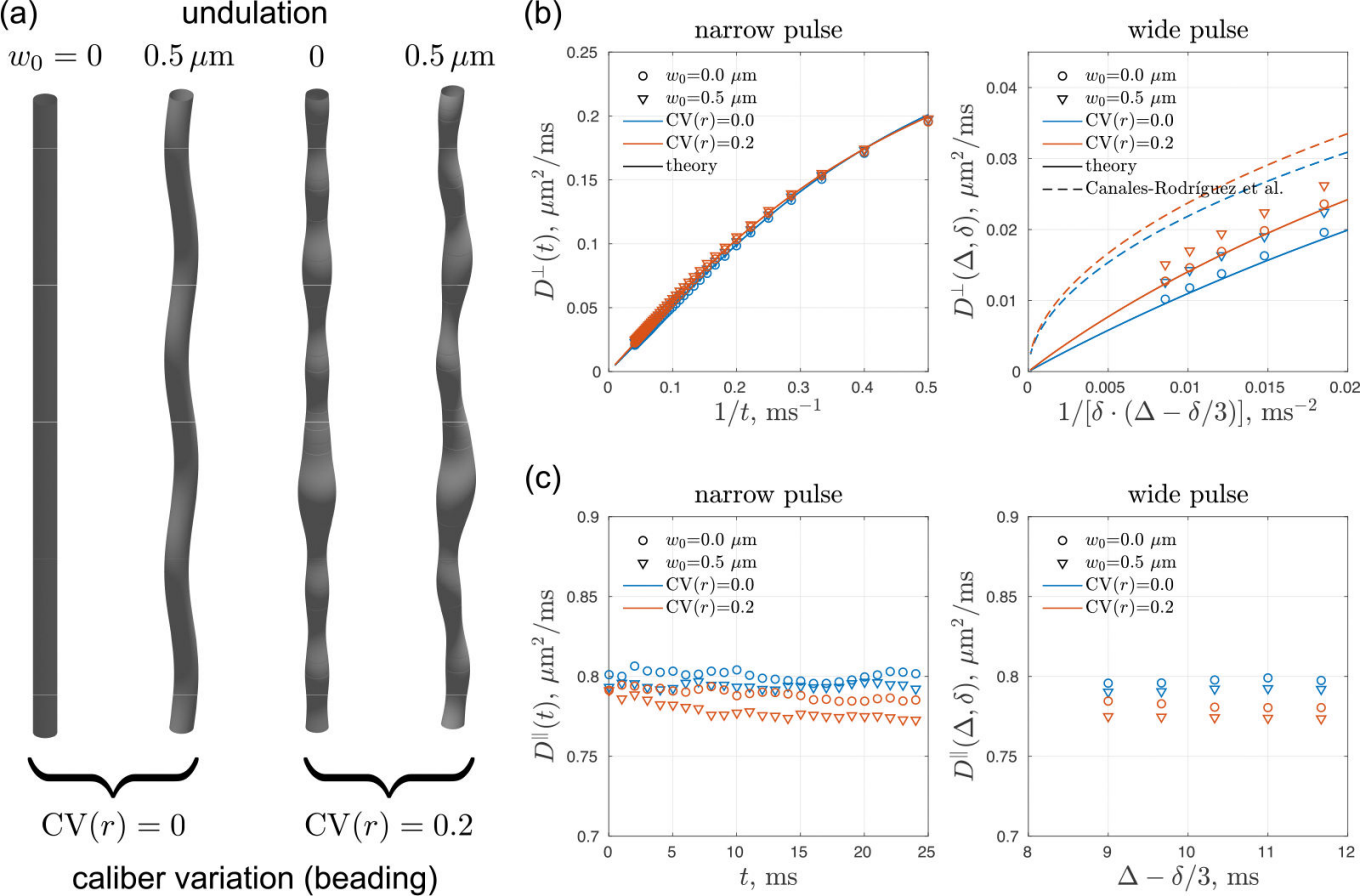
Monte Carlo simulation of radial diffusivity and axial diffusivity in myelin water of axons with caliber variations and undulations. (a) To explore the effect of realistic axonal shape on diffusion metrics, we generated four cylindrical shells with a finite thickness $l_{m}=12\;nm$ and varying caliber variations and undulations, tuned by coefficient of variation of radius CV(r) and undulation amplitude $w_{0}$, respectively. The undulation wavelength was fixed at 20 *μ*m. (b) Simulated radial diffusivity (data points) in cylindrical shells with either caliber variations or undulations is slightly higher than that in perfectly straight cylindrical shells. The effect of caliber variations on radial diffusivity can still be captured by the theory (solid curves) (2) for narrow-pulse and (6) for wide-pulse pulsed gradient sequences, whereas the effect of undulations cannot be predicted by current theory. In contrast, the alternative wide-pulse solution extended from the narrow-pulse one, i.e., (2) with t=Δ+δ, cannot predict the effect of caliber variations (dashed curves) [31]. (c) Simulated axial diffusivity (data points) in cylindrical shells with either caliber variations or undulations is slightly lower than that in perfectly straight cylindrical shells.

In simulations, we demonstrated that caliber variations and undulations had small effects on myelin water diffusion metrics, such as axial and radial diffusivities (Fig. 6). Given that the spherical mean signals (9) were parametrized only by axial and radial diffusivities of myelin water in MyCaliber model, we expected that realistic axonal shapes had very mild effect on ADM from myelin water diffusion. This was very different from that of ADM from intra-axonal water diffusion, where intra-axonal axial diffusivity was heavily affected by the caliber variations [67], and intra-axonal radial diffusivity was substantially affected by undulations [68].

As suggested by (13), the higher the SNR, the smaller (better) the resolution limit of ADM. For in vivo human brain scans of 2 mm isotropic resolution on clinical and preclinical scanners, the typical SNR from all signal contributions, including intra-/extra-axonal water and myelin water, is 20–50 in the brain white matter. To selectively measure myelin water diffusion signals, it is required to apply $T_{1}$ and/or $T_{2}$-selective acquisition with diffusion MRI, further reducing the SNR. Fortunately, recent advances in image denoising techniques have helped to boost the SNR by leveraging the data redundancy in diffusion MRI measurements, such as Marchenko-Pastur principal component analysis (MP-PCA) [39], [71], tensor PCA [40], threshold PCA [72], iterative Rician bias correction [73], and other deep-learning based methods [74], [75].

### A. Outlook

We performed simulations in perfectly straight cylindrical surfaces with no caliber variations (beadings) and undulations (tortuous axonal skeleton) [67], [68], [76], which may lead to under- and over-estimation of axonal diameters, respectively [49]. The presence of axonal varicosity (beading) has been observed in the pathology such as traumatic brain injury [61], [63], multiple sclerosis [62], and ischemia [64]. Studying the effect of these inhomogeneous micro-environments on ADM is beyond the scope of this study and should be explored in the future.

Furthermore, we demonstrated the applicability of ADM only for the regular pulsed gradient waveforms. Recent advances in general diffusion gradient waveforms, such as q-space trajectory imaging [77] and b-tensor encoding [78], have shown the potential in biophysical modeling of diffusion MRI. However, in [49], we showed that the application of general diffusion gradient waveforms (e.g., planar- and spherical-tensor encoding) did not help to improve the resolution limit of axonal diameter mapping using water diffusion of intra-axonal space, whose signal kernel was modeled as an axisymmetric diffusion tensor. Given that the myelin water diffusion of an myelinated axon segment was also modeled as an axisymmetric diffusion tensor (Section II-C) [31], we expected that general diffusion gradient waveforms may not help to improve the resolution limit of axonal diameter estimations.

Based on (13), the higher the diffusion gradient strength, the smaller (better) the resolution limit of ADM. In addition, the strong diffusion gradients help to further shorten the diffusion waveform as well as the echo time, leading to a higher SNR for attainment of resolutions better than what is currently achievable on routine clinical scanners [28], [29]. In this study, we evaluated the applicability of ADM using myelin water diffusion for the protocol accessible on the one-of-a-kind human Connectome 2.0 MRI scanner equipped with the maximal gradient strength $G_{\text{max}}=500\;mT/m$. Fortunately, recent advances in commercially available clinical and preclinical MR human scanners ($G_{\text{max}}=200\;mT/m$) [27], [58] potentially enable in vivo evaluation of axonal diameters using the same technique. This will need to be further tested in follow-up studies.

In this study, we only considered myelin water diffusion. To exclusively measure diffusion signals of myelin water, it is essential to apply $T_{1}$-manipulations [35], such as magnetization transfer and inversion recovery. Furthermore, to account for the very short $T_{2}$ value of myelin water, it is required to shorten the echo time by applying strong diffusion gradients to achieve sufficiently high diffusion weightings at the same time [28], [29].

**Fig. 7.**
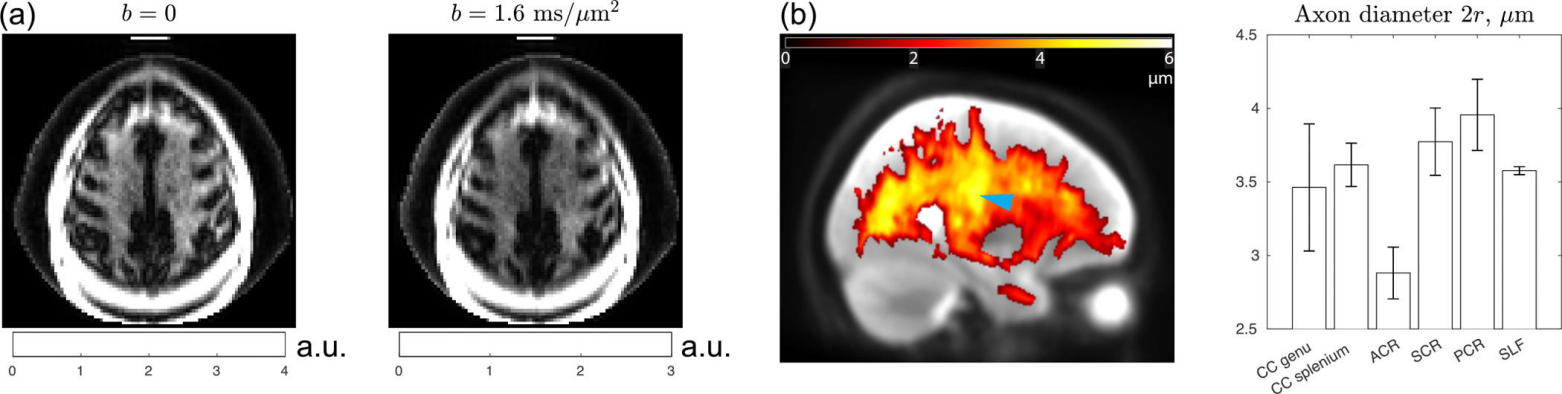
Myelin water selected diffusion MRI measurements and axon diameter mapping in brain white matter. To selectively measure myelin water diffusion signals, we performed diffusion MRI with inversion recovery and short repetition time to saturate signal contributions from (intra- and extra-) axonal water and cerebrospinal fluid. (a) The non-diffusion-weighted-image averaged over 68 repetitions (left) and spherical mean signal averaged over 80 directions at b-value = 1.6 ms/*μ*m^2^ (right) showed negligible partial volume effects from cerebrospinal fluid. The skull fat signal was noticeable due to incomplete fat saturation in images with low SNR. (b) Fitting MyCaliber model (9) and (6) to normalized spherical mean signal in white matter, we estimated axon diameter maps averaged over 3 healthy subjects (left) and showed thick axons in the corticospinal tract (blue arrow). The ROI analysis in JHU DTI white matter atlas (right) showed an anterior-to-posterior gradient (from ACR, SCR, to PCR) of axon diameters in the brain. The a.u. indicated the arbitrary units of the MRI signal. The error bar indicated the standard deviation of 3 subjects in each ROI. CC = corpus callosum; ACR, SCR, PCR = anterior, superior, and posterior corona radiata; SLF = superior longitudinal fasciculus.

To demonstrate the applicability of the proposed ADM from myelin water, we acquired the pilot data of myelin water diffusion MRI of three healthy subjects 23–30 years old, 3 males) on the 3T Connectome 2.0 MRI scanner (MAGNETOM Connectom.X; Siemens Healthineers, Forchheim) equipped with 500 mT/m gradient strength and 600 T/m/s slew rate [28], [29]. The volunteer provided informed consent prior to participation in the study, which was approved by the Institutional Review Board of Massachusetts General Brigham. We acquired diffusion-weighted-images (DWIs) by using a PG-STAIR-SE (pulsed-gradient short TR adiabatic inversion recovery prepared spin-echo) echo-planar-imaging sequence of diffusion time Δ=10.1ms and pulse width δ=3.5ms at 4 b-values = 0.6, 0.9, 1.2, and 1.6 ms/*μ*m^2^ with 80 directions per b-shell and interspersed b=0 images for every 5 DWIs. To selectively measure myelin water diffusion signals, we applied the adiabatic inversion recovery of inversion time of 117 ms and short repetition time of 250 ms to saturate (intra- and extra-) axonal water ($T_{1}∼1\;s$) [18], [19] and cerebrospinal fluid ($T_{1}∼4\;s$) [79], respectively. A short echo time of 21 ms was used to maintain the SNR. The complex-valued b=0 images and DWIs were processed based on the DESIGNER pipeline [40], [41], [80], [81]. We fitted the MyCaliber model (9) and (6) to normalized spherical mean signals (Fig. 7a) in each voxel of brain white matter, segmented by using SynthSeg [82], and estimated the axon diameter map (Fig. 7b) that showed thick axons in the corticospinal tract. Furthermore, we performed region-of-interest (ROI) analysis using the JHU DTI white matter atlas [83] (Fig. 7b) and displayed an anterior-to-posterior gradient of axon diameters in the brain. The PG-STAIR-SE sequence measured an axon diameter that was weighted more toward the outer diameter (Appendix D), whereas the classical PGSE sequence measured an axon diameter that was heavily weighted by the tail of inner diameter distribution [9], [23].

For ADM through myelin water diffusion, water exchange between myelin layers and irregular myelin geometry were treated as confounding factors and shown to be negligible in our simulations. These effects can be quantified through other techniques. For example, water exchange between myelin layers can be estimated by using magnetization transfer [55], and irregular axonal geometries can be evaluated by using diffusivity time-dependence due to structural disorder [84].

Finally, we did not consider MR contrast mechanisms other than diffusion in our simulations. For example, myelin water signals can be affected by magnetization transfer [35] and fiber-direction-dependent susceptibility effects [85], [86], [87], [88], [89], [90], [91], [92]. Susceptibility-induced mesoscopic field gradients depend on fiber orientation and local micro-geometry [93], [94], [95], and caliber-varying and undulating fibers amplify local susceptibility variation. These effects can introduce subtle orientation-dependent modulation of diffusion signals and may contribute additional variability in vivo. Disentangling these effects from myelin water diffusion requires additional measurements with longer scan times. In our in vivo imaging protocol, adiabatic inversion pulses with short repetition time suppressed long $T_{1}$ water, leaving myelin water as the dominant signal source. Under this single-compartment condition with only myelin water, normalizing each DWI by the non-DWI (b=0 image) removed both MT and $T_{2}$ weighting in the spherical mean signal, since energy deposition and $T_{2}$ decay were identical. As a result, diffusivity estimates were not biased by these mechanisms.

## Appendix A Time -Dependent Diffusion Transverse to a Circle

The diffusion around a circle of radius r can be considered as a 1-dimensional Gaussian diffusion with a periodic boundary condition. The narrow-pulse dMRI signal along x=rcosθ is a sum over Laplace eigenmodes $∼e^{in\theta +\lambda_{n}t}$ with n∈Z, eigenvalues $\lambda_{n}=-n^{2}/t_{c}$, and the correlation time $t_{c}$ defined in (2):

(15)
$$S=\sum_{n=-\infty }^{\infty }\oint \frac{d\theta \;d\theta^{\prime }}{(2\pi {)}^{2}}\;e^{iqr(cos\theta -cos\theta^{\prime })-in(\theta -\theta^{\prime })-n^{2}t/t_{c}},$$

where q is the diffusion wave vector. Decomposing $e^{iqr\;cos\theta }=\sum_{m=-\infty }^{\infty }i^{m}J_{m}(qr)e^{im\theta }$, where $J_{m}$ is the Bessel function of the first kind, one obtains [31], [34]

(16)
$$S=J_{0}^{2}(qr)+2\sum_{n=1}^{\infty }J_{n}^{2}(qr)e^{-n^{2}t/t_{c}}.$$

The Taylor expansion of signal (16) at low diffusion weighting $b=q^{2}t$ yields

(17)
$$S≃1-b\cdot \frac{r^{2}}{2t}\left(1-e^{-t/t_{c}}\right)+𝒪\left(b^{2}\right),$$

where the leading term 𝒪(b) is only contributed by the first two eigenmodes ($J_{0}^{2}$ and $J_{1}^{2}$) in (16). The definition $D^{⊥}(t)\equiv -{\left.\frac{1}{b}lnS\right|}_{b\to 0}$ yields the diffusivity (2) transverse to the circle.

For wide-pulse pulsed gradient sequence, we calculated the apparent diffusivity transverse to a 1-dimensional circle by using the Gaussian phase approximation [2], [23], [43], [45]. To begin with, we calculated the instantaneous diffusivity [2], [84] transverse to a circle using the second order cumulant of diffusion displacement $\left\langle x^{2}\right\rangle$ in (1) and (2),

$$D_{inst}^{⊥}\left(t\right)\equiv \frac{1}{2}\partial_{t}\left\langle x^{2}\right\rangle =\frac{1}{2}D_{0}\cdot e^{-\frac{t}{t_{c}}},$$

yielding the frequency (ω)-dependent dispersive diffusivity [2], [84],

(18)
$$\begin{array}{l} {𝒟}^{⊥}(\omega )\;\equiv -i\omega \int_{0}^{\infty }\;dte^{i\omega t}D_{inst}^{⊥}(t)\\ \;=\frac{1}{2}D_{0}\cdot \frac{-i\omega t_{c}}{1-i\omega t_{c}}. \end{array}$$

Under Gaussian phase approximation [2], [23], [45], the normalized diffusion signal can be approximated by

(19)
$$-\ln S≃\int_{-\infty }^{\infty }\frac{d\omega }{2\pi }{𝒟}^{⊥}\left(\omega \right){\left|q_{\omega }\right|}^{2},$$

where $q_{\omega }$ is the Fourier transform of diffusion wave vector $q(t)=\gamma \int \;G\left(t^{\prime }\right)dt^{\prime },\gamma$, is the gyromagnetic ratio of water proton, and G is the diffusion gradient strength. For a wide-pulse pulsed gradient sequence of inter-pulse duration ∆ and pulse width δ, we have [23], [96]

(20)
$$q_{\omega }=\frac{\gamma G}{(i\omega {)}^{2}}\left(e^{i\omega \delta }-1\right)\left(e^{i\omega \Delta }-1\right).$$

Substituting (18) and (20) into (19), we obtained the apparent diffusivity transverse to a circle of wide-pulse pulsed gradient sequence (6) based on the definition $D^{⊥}(\Delta ,\delta )\equiv -{\left.\frac{1}{b}lnS\right|}_{b\to 0}$, with the diffusion weighting b in (10).

## Appendix B Resolution Limit of Axon Radius Estimation

To estimate the resolution limit, we calculate the differences between normalized spherical mean signals of myelin water (9) in axons of finite radius r and infinitely thin axons [46]:

(21)
$$\Delta S={\left.S(r)\right|}_{r=0}-S\left(r\right)=\left[1-\exp\left(-bD^{⊥}\right)\right]\cdot h\left(A\right).$$

For thin axons of radii r∼1μm, the myelin water RD in narrow-pulse limit is $D^{⊥}\left(t\right)∼0.05\;\mu m^{2}/ms$ at diffusion time t=10ms based on long-time solution (3). This $D^{⊥}$ value is much smaller the myelin water AD $D^{\|}≲D_{0}∼0.8\;\mu m^{2}/ms$, suggesting

(22)
$$A≃bD^{\|},\;D^{⊥}\ll D^{\|}.$$

Further, the small value of $D^{⊥}$ justifies the approximation $exp\left(-bD^{⊥}\right)≃1-bD^{⊥}$, leading to

(23)
$$\Delta S≃bD^{⊥}\cdot h(A),\;bD^{⊥}\ll 1.$$

To observe significant differences between diffusion signals of finite-radius and zero-radius axons, the z-score of the signal differences has to be larger than the z-threshold $z_{\alpha }$ at the significance level α [46]:

(24)
$$z\left(\Delta S\right)=\frac{\Delta S}{\sigma \sqrt{n_{g}}}\geq z_{\alpha },$$

where the normalized noise level is defined by σ≡1/SNR, and $n_{g}$ is the number of gradient direction per b-shell.

For narrow-pulse pulsed gradient sequence, substituting (3), (22), and (23) into (24) yields the resolution limit (12) of axon radius estimation.

Similarly, for thin axons of radii r∼1μm, the myelin water RD in wide-pulse limit is $D^{⊥}\left(\Delta ,\delta \right)∼0.019\;\mu m^{2}/ms$ at Δ=13ms and δ=6ms based on (7), again justifying the approximation in (22) and (23). For wide-pulse pulsed gradient sequence, substituting (7), (22), and (23) into (24) yields the resolution limit (13) of the axon radius estimation.

## Appendix C Surface -to-Volume Ratio in Each Compartment

The water exchange rate R from one compartment to another is related with the membrane permeability κ and surface-to-volume ratio (S/V) [97]:

$$R=\kappa \cdot \frac{S}{V}.$$

In other words, the larger the surface-to-volume ratio, the stronger the exchange effect.

For a myelin layer of radius r and thickness $l_{m}\ll r$, its surface-to-volume ratio is

$${\left(\frac{S}{V}\right)}_{myelin}≃\frac{4\pi r}{2\pi r\cdot l_{m}}=\frac{2}{l_{m}},$$

which is roughly 166.6 *μ*m^−1^ for a myelin layer of thickness $l_{m}=12\;nm$. For an intra-axonal space of inner radius $r_{i}$, its surface-to-volume ratio is

$${\left(\frac{S}{V}\right)}_{\text{in}\;}=\frac{2}{r_{i}},$$

which is less than 2 *μ*m^−1^ for axons thicker than 1 *μ*m in inner radius. Finally, for an extra-axonal space in-between axons of inner radius $r_{i}$ and outer radius $r_{o}$, its surface-to-volume ratio is

$${\left(\frac{S}{V}\right)}_{ex}=\frac{2\pi r_{o}\cdot N}{f_{ex}\cdot V}=\frac{2}{r_{i}}\cdot \frac{f_{in}}{gf_{ex}},$$

where N is the number of axons in an MR voxel of volume V, $f_{\text{in}}$ and $f_{\text{ex}}$ are the intra- and extra-axonal volume fractions, respectively, and g-ratio is the ratio of inner to outer axon radii, i.e., $g=r_{i}/r_{o}$. We use the relation $f_{\text{in}}=N\cdot \pi r_{i}^{2}/V$ to derive the above equation. The typical values of tissue parameters are $f_{\text{in}}=0.6,$ $f_{\text{ex}}=0.25$ (with the consideration of myelin water fraction ~ 0.15), and g=0.7 [15], [98], [99], yielding an estimate of $(S/V{)}_{\text{ex}}$ less than 6.9 *μ*m^−1^ for axons thicker than 1 *μ*m in inner radius. Based on the above analysis, we assume that the water exchange effect is dominated by the exchange between myelin water layers with very high surface-to-volume ratio.

## Appendix D Interpretation of Axon Diameter Estimation

We aimed to better interpret the estimated axon diameter from myelin water diffusion, measured by using PG-STAIR-SE sequence. For simplicity, we assumed that the axon diameter distribution in a voxel can be approximated as a Gamma distribution [3], [53], [100], [101], and all axons have a fixed g-ratio (ratio of inner to outer diameter). The n-th order moment of myelin sheath radii r for a single axon j of inner radius $r_{i}$, outer radius $r_{o}$, and g-ratio $g=r_{i}/r_{o}$ can be expressed as

$${\left\langle r^{n}\right\rangle }_{j}=\frac{\int_{r_{i}}^{r_{o}}\;r^{n}\;dr}{\int_{r_{i}}^{r_{o}}\;\;dr}=\frac{r_{i}^{n}}{n+1}\cdot \frac{g^{-(n+1)}-1}{g^{-1}-1}.$$

The moment of inner axon radius in a Gamma distribution is given by

(25)
$$\left\langle r_{i}^{n}\right\rangle =\frac{\theta^{n}\cdot \Gamma (\kappa +n)}{2^{n}\cdot \Gamma (\kappa )},$$

where κ and θ are shape and scale parameters of Gamma distribution of inner axon diameter, and Γ is Euler’s Gamma function. Given that the g-ratio of all axons were assumed to be the same for simplicity, we combined the above two equations and obtained the overall moment of myelin sheath radii, given by

$$\left\langle r^{n}\right\rangle =\frac{\theta^{n}\cdot \Gamma (\kappa +n)}{2^{n}\cdot \Gamma (\kappa )}\cdot \frac{1}{n+1}\cdot \frac{g^{-(n+1)}-1}{g^{-1}-1}.$$

Substituting into various definitions of effective diameters for myelin water diffusion, such as mean diameter 2⟨r⟩, volume-weighted averaged diameter $2r_{\text{vwa}}$ (5), effective diameter of narrow-pulse sequence $2r_{\text{eff,NP}}$ (4), and effective diameter of wide-pulse sequence $2r_{\text{eff,WP}}$ (8), we obtained

$$2\langle r\rangle =\kappa \theta \cdot \frac{g^{-1}+1}{2},$$

$$2r_{vwa}=\kappa \theta \cdot \left(1+\kappa^{-1}\right)\cdot \frac{2}{3}\cdot \frac{g^{-2}+g^{-1}+1}{g^{-1}+1},$$

$$2r_{eff,NP}=\kappa \theta \cdot {\left[\left(1+\kappa^{-1}\right)\left(1+2\kappa^{-1}\right)\cdot \frac{g^{-2}+1}{2}\right]}^{1/2},$$

$$2r_{eff,WP}=\kappa \theta \cdot {\left[\prod_{m=1}^{4}\;\left(1+m\cdot \kappa^{-1}\right)\cdot \frac{g^{-4}+g^{-2}+1}{3}\right]}^{1/4}.$$

Here we assumed that inner diameter’s standard deviation $\sqrt{\kappa }\theta$ was half of its mean $2{\overset{‾}{r}}_{i}=\kappa \theta$ as in our simulations, i.e., κ=4, and the g-ratio was around 0.7 [53], [100], [101]. Then we obtained the effective diameter of myelin water diffusion:

$$2\langle r\rangle \approx 1.21\times 2{\overset{‾}{r}}_{i}=0.85\times 2{\overset{‾}{r}}_{o},$$

$$2r_{vwa}\approx 1.53\times 2{\overset{‾}{r}}_{i}=1.07\times 2{\overset{‾}{r}}_{o},$$

$$2r_{eff,NP}\approx 1.69\times 2{\overset{‾}{r}}_{i}=1.18\times 2{\overset{‾}{r}}_{o},$$

$$2r_{eff,WP}\approx 1.99\times 2{\overset{‾}{r}}_{i}=1.39\times 2{\overset{‾}{r}}_{o},$$

where the outer diameter’s mean was $2{\overset{‾}{r}}_{o}=2{\overset{‾}{r}}_{i}/g$. This example showed that the volume-weighted averaged diameter $2r_{\text{vwa}}$ was close to the outer axon diameter. Given that the axon diameter estimated by the myelin water diffusion coincided with the volume-weighted average in our simulations, we concluded that the axon diameter estimated by the PG-STAIR-SE could be a surrogate of the outer axon diameter.
